# A randomized approach to speed up the analysis of large-scale read-count data in the application of CNV detection

**DOI:** 10.1186/s12859-018-2077-6

**Published:** 2018-03-01

**Authors:** WeiBo Wang, Wei Sun, Wei Wang, Jin Szatkiewicz

**Affiliations:** 1 Department of Computer Science, University of North Carolina at Chapel Hill, 201 S. Columbia St., Chapel Hill, 27599-3175 USA; 2 Biostatistics Program, Fred Hutchinson Cancer Research Center, 1100 Fairview Ave N, Seattle, 19024 USA; 3 Department of Computer Science, University of California, Los Angeles, 580 Portola Plaza, Los Angeles, 90095-1596 USA; 4 Department of Genetics, University of North Carolina at Chapel Hill, 120 Mason Farm Road, Chapel Hill, 27599-7264 USA

**Keywords:** Bioinformatic, Computational biology, Next-generation sequencing

## Abstract

**Background:**

The application of high-throughput sequencing in a broad range of quantitative genomic assays (e.g., DNA-seq, ChIP-seq) has created a high demand for the analysis of large-scale read-count data. Typically, the genome is divided into tiling windows and windowed read-count data is generated for the entire genome from which genomic signals are detected (e.g. copy number changes in DNA-seq, enrichment peaks in ChIP-seq). For accurate analysis of read-count data, many state-of-the-art statistical methods use generalized linear models (GLM) coupled with the negative-binomial (NB) distribution by leveraging its ability for simultaneous bias correction and signal detection. However, although statistically powerful, the GLM+NB method has a quadratic computational complexity and therefore suffers from slow running time when applied to large-scale windowed read-count data. In this study, we aimed to speed up substantially the GLM+NB method by using a randomized algorithm and we demonstrate here the utility of our approach in the application of detecting copy number variants (CNVs) using a real example.

**Results:**

We propose an efficient estimator, the randomized GLM+NB coefficients estimator (RGE), for speeding up the GLM+NB method. RGE samples the read-count data and solves the estimation problem on a smaller scale. We first theoretically validated the consistency and the variance properties of RGE. We then applied RGE to GENSENG, a GLM+NB based method for detecting CNVs. We named the resulting method as “R-GENSENG". Based on extensive evaluation using both simulated and empirical data, we concluded that R-GENSENG is ten times faster than the original GENSENG while maintaining GENSENG’s accuracy in CNV detection.

**Conclusions:**

Our results suggest that RGE strategy developed here could be applied to other GLM+NB based read-count analyses, i.e. ChIP-seq data analysis, to substantially improve their computational efficiency while preserving the analytic power.

**Electronic supplementary material:**

The online version of this article (10.1186/s12859-018-2077-6) contains supplementary material, which is available to authorized users.

## Background

High-throughput sequencing (HTS) has been used in a range of genomic assays in order to quantify the amount of DNA molecules (DNA-seq), or genomic regions enriched for certain biological processes (ChIP-seq, DNase-seq, FAIRE-seq) [1–4]. Typically, sequencing reads are first aligned to the reference genome and a summary metric is then defined per counting unit (e.g., a window) and used as a method of quantification in the subsequent comparative analysis. In DNA-seq, windowed read counts, defined as the number of reads falling into consecutive windows of fixed size tiling the genome (e.g., 200bp, 500bp), are used to detect regions of copy number changes (i.e., CNVs such as deletions and duplications) [5–11]. Similarly, windowed read counts are used in ChIP-seq, DNase-seq, and FAIRE-seq to detect regions with strong local aggregations of mapped reads, referred to as “enriched regions" [12, 13]. These windowed read counts are by nature a series of counts, for which the negative-binomial (NB) distribution has been shown to be the suitable distribution in statistical modeling [10, 14–16]. The NB model is flexible for modeling genomic read-count data because its dispersion parameter allows a larger variance and therefore is less restrictive than the Poisson distribution. Further, via GLMs [17], the NB model provides a powerful framework simultaneously to account for confounding factors (e.g., genomic GC content and mappability) and to determine the true relationships between read-count signals and biological factors [10].

A large number of statistical methods and software tools have been developed to create GLM+NB models for analyzing genomic read-count data. For example, GENSENG [10] was developed for detecting CNVs using DNA-seq; ZINBA [16] for detecting enriched regions using ChIP-seq, DNase-seq, or FAIRE-seq. However, while statistically powerful, GLM+NB methods encounter a big data problem [18] when applied to whole-genome windowed read count data with tens of millions of windows. Such applications include detecting CNV from whole-genome DNA-seq data [8, 10], detecting enrichment peaks from whole-genome ChIP-seq data [19], and finding association between histone modification or open chromatin with DNA sequence content [20].

The iterative reweighed least square (IRLS) algorithm is the standard approach used to fit GLMs [21]. The complexity of IRLS algorithm is quadratic with respect to the number of coefficients, and IRLS needs to be run multiple times until it converges. The large computation cost of GLM hinders the computational efficiency of the GLM+NB methods when applied to large scaled windowed read-count data. The popular methods to tackle this problem include sampling (i.e. randomized algorithms) and distributed computing. Sampling based methods intend to obtain analysis results comparable to full data sets analysis with smaller computational cost by analyzing only a subset of the full data sets [22]. The distributed computing based methods intend to perform the analysis in parallel on distributed computation environment. Although the distributed computation environment is not uncommon in many academic institutes, it is expensive to maintain a cluster and the distributed computation environment is not easily accessible to many other researchers, such as those who work in companies. In this study, we aimed to improve substantially the computational efficiency of the GLM+NB methods by using a randomized algorithm.

The randomized algorithm is a general computational strategy that has been widely studied by multiple disciplines, such as theoretic computer science and numerical linear algebra [23]. The basic idea is to sample a subset of rows or columns from the input data matrix and solve the problem on the sampled data with its much reduced and manageable scale. The randomized algorithm is asymptotically faster than existing deterministic algorithms and is faster in numerical implementation in terms of clock time [23, 24]. This feature is especially appealing with respect to the problem of GLM+NB methods because of the quadratic computational complexity of the IRLS algorithm [22, 25–31]. The choice of sampling strategies used to select the data subset is important to the performance of the randomized algorithm. Recent analyses have evaluated the algorithmic and statistical properties of various sampling strategies under regression models, including uniform sampling and weighted sampling (a.k.a. probability sampling) [22, 32]. Uniform sampling selects rows from the input data matrix uniformly at random, whereas weighted sampling selects rows with probability proportional to its empirical statistical leverage score of the matrix. While both uniform and weighted sampling strategies provide unbiased estimates of the regression coefficients, the variance properties may vary depending on their applications [22]. In this study, we introduce RGE (randomized GLM+NB coefficients estimator) as a viable approach for accelerating the GLM+NB-based read-count analysis. In the application of RGE for CNV detection, we have chosen the weighted sampling strategy, based on our empirical evidence that it yields smaller estimation variance than uniform sampling.

To illustrate the utility of RGE, we used a GLM+NB-based CNV detection method GENSENG [10] as an example and named the resulting RGE-GENSENG as “R-GENSENG”. In a genome sequencing experiment, the relationship between the windowed read-counts and the underlying copy numbers is distorted by various sources of bias. In order to accurately detect CNVs, the effects of biases must be corrected and, if bias correction is integrated into read-count analysis, the improvement in CNV detection is more substantial than if the bias correction is otherwise integrated [8, 10]. GENSENG implements a hidden Markov model (HMM) and the GLM+NB method to integrate bias correction and read-count analysis in a one-step procedure. In GENSENG, the HMM emission probability describes the likelihood of the observed read-count data and is computed as a mixture of uniform distribution and the NB regression model (a form of GLM); therefore, this method simultaneously accounts for multiple confounding factors (e.g., GC content and mappability) by including them as regression covariates and the NB dispersion parameter accounts for the unknown sources of bias.

As described below, we first evaluated the consistency and the variance properties of RGE. We concluded that RGE is a consistent GLM+NB regression estimator and that its implementation using a weighted sampling strategy yields smaller regression coefficients and estimated variance than those obtained using a uniform sampling strategy. We then performed simulation and real-data analysis to evaluate R-GENSENG and to compare it with the original GENSENG. We concluded R-GENSENG is ten times faster than the original GENSENG while maintaining GENSENG’s accuracy in CNV detection. Our results suggest that RGE and the strategy developed in this work could be applied to other GLM+NB based read-count analyses to substantially improve their computational efficiency while preserving the analytic power.

## Methods

In this section, we first introduce RGE’s critical statistical properties concerning consistency and variance and then we introduce R-GENSENG. We evaluated the consistency of RGE because RGE uses a subset of the data points to estimate NB regression coefficients. We required the sampling strategy applied in RGE yielding a non-singular sampled matrix. Given such a sampling strategy we show that, the resulting estimates converge in probability to the true coefficient values as the number of data points used increasing indefinitely. We evaluated the variance of RGE because RGE applies a weighted sampling strategy to select the subset of data and we wanted to investigate the effects of the sampling strategy on the variance. Below we show that a weighted sampling approach yields a smaller estimated variance than does a uniform sampling strategy.

### The consistency of RGE

Following notations, we summarize the main theory in Theorem 1 and defer the detailed proof to the [see Additional file 1].

We denote by $\mathbf {X} \in \mathbb {R}^{n \times p}$ the design matrix that is composed of *n* rows and *p* columns, and $\mathbf {y} \in \mathbb {R}^{n}$ the *n*-dimensional response vector. Let **x**_*j*_=(*x*_1*j*_,...,*x*_*nj*_)^*T*^ be the *j*-th column of **X**, and $x_{i,j} \in \mathbb {R}$ be the element at the *i*-th row and *j*-th column of **X**. Let **X**^*T*^ be the transpose of **X**. Let ∥**v**∥_*∞*_ be the maximum absolute value of the elements of a vector **v**.

We consider the response vector **y** with all its elements independently generated from an exponential family distribution with the density function

$$\begin{aligned} f_{n}\left(\mathbf{y};\mathbf{X},\boldsymbol{\beta}\right) \equiv \prod_{i=1}^{n}f_{0}\left(y_{i};\theta_{i},\varphi\right) = \prod_{i=1}^{n} \left\{\exp \left [ \frac{y_{i}\theta_{i}-b(\theta_{i})}{\varphi} +c(\thinspace{y}_{i}, \varphi)\right ] \right\} \end{aligned} $$

where { *f*_0_(*y*_*i*_;*θ*_*i*_,*φ*)} is a distribution in the exponential family with canonical parameter *θ*_*i*_ and GLM dispersion parameter *φ*>0.

A negative binomial distribution is in the exponential family when its over-dispersion parameter *ϕ* is fixed. Let $\eta _{i} = x_{i}^{T}\boldsymbol {\beta } = g(\mu _{i}) = E(y_{i})$, where *g* is a link function. Given a log link function, *η*_*i*_=*g*(*μ*_*i*_)= log(*μ*_*i*_), the unknown *p*-dimensional vector of regression coefficients ***β***=(*β*_1_,...,*β*_*p*_)^*T*^ in the negative binomial model can be estimated with the IRLS procedure. In step *t* of the procedure the parameter ***β***^(*t*)^ is updated with the Fisher scoring equation

1 $$ \left[\mathbf{X}^{T} W^{(t-1)} \mathbf{X}\right] \boldsymbol{\beta}^{(t)} = \mathbf{X}^{T} W^{(t-1)} \left[ \mathbf{X} \boldsymbol{\beta}^{(t-1)} + \boldsymbol{\zeta} \right],  $$

where *W* is a diagonal *n*×*n* matrix, with the *i*-th diagonal element *w*_*i*_=*μ*_*i*_/(1+*μ*_*i*_*ϕ*), ***ζ*** is a vector of length *n*, with the *i*-th element ***ζ***_*i*_=(*y*_*i*_−*μ*_*i*_)/*μ*_*i*_. The NB over-dispersion parameter *ϕ* is fixed in this step. The details of the GLM-NB estimation are described in Additional file 1, page 1, Section 1.1. In each step, after ***β*** is estimated, the NB over-dispersion parameter can be then estimated with fixed ***β***. The estimation of *ϕ* with fixed coefficients is described in Additional file 1, page 9, Section 2.4.8. The randomized approach applies when coefficients are estimated by fixing the NB over-dispersion parameter *ϕ*.

Let ***β***_0_=(*β*_01_,...,*β*_0*p*_) be the coefficients of Eq. (1) updated with the full data, we will show that there exists a solution that is inside the hypercube of ***β***_0_ using sampled data.

Let the sampling indicator for the *i*-th entry, *i*=1,...,*n* be

$$ m_{i} = \left\{ \begin{array}{lc} 1 & \text{if}\ i\text{-th entry is sampled,} \\ 0 & \text{otherwise}. \end{array} \right. $$

For equation

2 $$ f(\boldsymbol{\beta}) = {\bar{\mathbf{X}}^{\mathbf{T}}}\left(\mathbf{m} \circ \bar{\mathbf{X}}\boldsymbol{\beta}\right) - {\bar{\mathbf{X}}^{\mathbf{T}}(\mathbf{m }\circ \bar{\mathbf{y}})},  $$

where ${\bar {\mathbf {X}}}=\mathbf {X}W_{(t-1)}^{1/2}$, ${\bar {\mathbf {y}}}= W_{(t-1)}^{1/2}\mathbf {z}$ is a known vector of length *n* with *z*_*i*_=*x*_*i*_***β***^***(t−1)***^+(*y*_*i*_−*μ*_*i*_)/*μ*_*i*_, ∘ is the Hadamard (component wise) product, we have

#### **Theorem 1**

For sufficient large *n*, there exists a solution $\hat {\boldsymbol {\beta }} \in \mathbb {R}^{p}$ for Eq. *(2)* of ${\bar {\mathbf {X}}^{\mathbf {T}}}\left ({\mathbf {m} \circ \bar {\mathbf {X}}\boldsymbol {\beta }}\right)-{\bar {\mathbf {X}}^{\mathbf {T}}(\mathbf {m} \circ \bar {\mathbf {y}})}=0$ inside the hypercube

$$\mathcal{N}_{0}=\left\{\boldsymbol{\delta} \in \mathbb{R}^{p} : \| \boldsymbol{\delta} - \boldsymbol{\beta}_{0} \|_{\infty} \leq d_{n}=O(n^{-\gamma_{0}} \sqrt{\log{n}})\right\}, $$

assuming the sampled matrix ${\bar {\mathbf {X}}^{\mathbf {T}}}\text {diag}\left (\mathbf {m}\right){\bar {\mathbf {X}}}$ is not singular, $d_{n} \equiv 2^{-1} \min _{1\leq j \leq p}\left \{|\beta _{0j}|\right \}=O\left (n^{-\gamma _{0}}\sqrt {\log {n}}\right)$ for some *γ*_0_∈(0,1/2).

### The variance of RGE

RGE applies a weighted sampling strategy since this approach potentially yields an estimated variance which is smaller than that obtained using uniform sampling. Using a one-way NB regression model as an example, we evaluated and compared the inverses of the Fisher information matrix between RGE’s weighted sampling and uniform sampling.

The co-variance matrix of the maximum likelihood estimator (MLE) ***β*** is the inverse of the Fisher information matrix $-E\left (\frac {\partial ^{2} \ell }{\partial \boldsymbol {\beta }^{2}}\right)$. The Fisher information matrix is a *p*×*p* matrix, and its (*j*,*k*)-th element equals to

$$-E\left(\frac{\partial^{2} \ell}{\partial \beta_{j} \partial \beta_{k}}\right)= \sum_{i=1}^{n}\frac{\mu_{i}^{2}}{\text{Var}(y_{i})}x_{ij}x_{ik}, $$

if the link function is the log function.

We illustrate the method using a simple one-way NB regression model: log(*μ*)=*β*_0_+*β*_1_(*CN*), where the link function is the log link function, *μ* is the mean value of read-count, *β*_0_ is the intercept, and *β*_1_ is the coefficient of the copy number *CN*. The *CN* measurements take three values: 0 for deletions, 1 for copy number neutral, and 2 for duplications. This model includes the general characteristics of the read-count analysis: a biological factor (e.g., copy number in CNV detection, or chromatin state in ChIP-seq) with three states including one state representing the baseline (e.g., copy number neutral) and two states representing the bidirectional differences from the baseline (e.g., deletions and duplications). In real-life applications, it is important to account for potential confounding factors (such as mappability, GC content etc.) in read count analysis [10, 16]. Confounding factors can be incorporated into this model by fitting all those terms together and then using them as the offset (i.e. fixing the coefficients of those terms).

Under this regression model, the Fisher information matrix is a 2×2 matrix including the intercept. The (1,1) element is $\sum _{i=1}^{n}\frac {1}{\text {Var}(y_{i})}$, the (1,2) and the (2,1) elements are $\sum _{i=1}^{n}\frac {1}{\text {Var}(y_{i})}x_{i}$, and the (2,2) element is $\sum _{i=1}^{n}\frac {1}{\text {Var}(y_{i})}x_{i}^{2}$, where *x*_*i*_ is the copy number of the *i*-th observation. The inverse of a 2×2 matrix could be obtained analytically. Here we are interested in the variance of the coefficient of the copy number, which is the (2,2) element of the inverse matrix. Define *p*_1_ as the probability of deletion event happening, *p*_2_ as the probability of copy number neutral happening, and *p*_3_ as the probability of duplication happening. With the log link function, the (2,2) element equals

3 $$ \frac{p_{1}r+p_{2}s+p_{3}t}{n \left(p_{1}p_{2}rs+4p_{1}p_{3}rt+p_{2}p_{3}st\right)},  $$

where $r=\left (e^{-\beta _{0}}+\phi \right)^{-1}$, $s=\left (e^{-\beta _{0}-\beta _{1}}+\phi \right)^{-1}$, and $t=\left (e^{-\beta _{0}-2\beta _{1}}+\phi \right)^{-1}$.

From Eq. (3) we find that when the uniform sampling is applied, *p*_1_,*p*_2_ and *p*_3_ would be the same in the sampled rows, but *n* would be smaller depending on the size of the sample. As a result, the variance would become larger. For example, if we uniformly sample 10% of all rows, the variance would be 10 times larger. Thus, the coefficients estimated from the sampled data have larger variances than using the full data.

We next compare the uniform sampling strategy with the weighted sampling strategy used in RGE by finding the minimum solution of Eq. (3) (i.e., the distribution of *p*_1_,*p*_2_ and *p*_3_ in the sampled data which yielded a minimum variance given the same sample size). We list below the Karush-Kuhn-Tucker (KKT)-conditions for minimizing Eq. (3), subject to constraints. First, the objective function under the KKT-conditions is

$$\begin{aligned} &\frac{p_{1}r+p_{2}s+p_{3}t}{n \left(p_{1}p_{2}rs+4p_{1}p_{3}rt+p_{2}p_{3}st\right)}\\ &\qquad\,\,+\lambda\left(1-p_{1}-p_{2}-p_{3}\right)-\mu_{1}p_{1}-\mu_{2}p_{2}-\mu_{3}p_{3}, \end{aligned} $$

where *λ* and *μ*_1_, *μ*_2_, and *μ*_3_ are KKT multipliers. And the necessary conditions for the minimum solution are

*Stationarity*

$$\begin{array}{rcl} \frac{r\left(p_{2}s+2p_{3}t\right)^{2}}{n\left(p_{1}p_{2}rs+4p_{1}p_{2}rt+p_{2}p_{3}st\right)^{2}} & = & \lambda + \mu_{1}, \\ \frac{s\left(p_{1}r-p_{3}t\right)^{2}}{n\left(p_{1}p_{2}rs+4p_{1}p_{2}rt+p_{2}p_{3}st\right)^{2}} & = & \lambda + \mu_{2}, \\ \frac{t\left(p_{2}s+2p_{1}r\right)^{2}}{n\left(p_{1}p_{2}rs+4p_{1}p_{2}rt+p_{2}p_{3}st\right)^{2}} & = & \lambda + \mu_{3}. \end{array} $$

*Primal feasibility and Dual feasibility*

$$\begin{array}{c} p_{1}+p_{2}+p_{3}=1, \\ p_{1} \ge 0, p_{2} \ge 0, p_{3} \ge 0, \\ \mu_{1} \ge 0, \mu_{2} \ge 0, \mu_{3} \ge 0. \end{array} $$

*Complementary slackness*

$$\begin{array}{c} \mu_{1}p_{1} = 0, \mu_{2}p_{2} = 0, \mu_{3}p_{3} = 0. \end{array} $$

Three possible solutions satisfy the KKT conditions.

$$\begin{array}{l} Solution 1 \\ p_{1}=0, p_{2}=\frac{\sqrt{st}}{\sqrt{st}+s}, p_{3}=\frac{\sqrt{s}}{\sqrt{s}+\sqrt{t}}, \\ \text{objective function}=\frac{\left(\sqrt{1/s}+\sqrt{1/t}\right)^{2}}{n} \\ \end{array} $$

$$\begin{array}{l} Solution 2 \\ p_{1}=\frac{\sqrt{t}}{\sqrt{r}+\sqrt{t}}, p_{2}=0, p_{3}=\frac{\sqrt{rt}}{\sqrt{rt}+t}, \\ \text{objective function}=\frac{\left(\sqrt{1/r}+\sqrt{1/t}\right)^{2}}{4n} \\ \end{array} $$

$$\begin{array}{l} Solution 3 \\ p_{1}=\frac{\sqrt{s}}{\sqrt{r}+\sqrt{s}}, p_{2}=\frac{\sqrt{rs}}{\sqrt{rs}+s}, p_{3}=0, \\ \text{objective function}=\frac{\left(\sqrt{1/r}+\sqrt{1/s}\right)^{2}}{n} \\ \end{array} $$

The objective function introduced above describes the scale of the inverse of the Fisher information matrix (i.e., the scale of the estimated variance). We thus want to know when the minimal solution of the objective function could be achieved. Within the setting, log(*μ*)=*β*_0_+*β*_1_(*CN*), where *CN* is the copy number from 0,1,2. In this case, when *CN*=0 (deletion), *β*_0_= log(*μ*), where *μ* is the expected read count for copy deletion, thus *β*_0_≥0. The read count will increase with the copy number in a linear manner (i.e., the read count of the copy number two region should be about twice the read count of the copy number one region), which suggests that the coefficient for CN *β*_1_ should be close to 1. Given *β*_0_≥0 and *β*_1_≃1, we have 1/*r*<1/*s*<1/*t*, and it is straightforward to see solution 3 is smaller than solution 1. We next compare solution 2 with solution 3. With a reasonable *μ*=0.1, we numerically solve the equation $\frac {\big (\sqrt {1/r}+\sqrt {1/t}\big)^{2}}{4}<\left (\sqrt {1/r}+\sqrt {1/s}\right)^{2}$ using the symbolic equation function in Matlab and conclude that solution 2 is the minimal solution. In solution 2, *p*_2_=0, which means that the variances obtained using sampled data will be minimized when only the rows representing CNVs are sampled.

The variance studies above show that (1) the regression coefficients estimated from the sampled data have a larger variance than using the full data; (2) the variances using the sampled data will be minimized when only the rows representing true CNVs (“CNV-rows" hereafter) are sampled. In the CNV detection problem, we do not have information regarding which rows are CNV-rows, but we can obtain the probability that each row represents a true CNV given the observed read-count data (e.g., the hidden Markov model posterior probability computed from GENSENG). Recent surveys of genetic variation found that there are >1000 CNVs in the human genome, accounting for ∼4 million bp or 0.1% of genomic difference at the nucleotide level [5, 33–35]. We therefore expect that CNV-rows are rare (<1%) in the input read-count data matrix. By assigning higher sampling probability to rows with higher probability of being CNV-rows, we would sample more CNV-rows than we would by using uniform sampling with equal probabilities. Consequently, we expect that this weighted sampling (weighted by the HMM posterior probability of a specific row being a CNV-row) would yield smaller variances of the coefficient estimates than a uniform sampling approach would obtain. We thus have chosen to use a weighted sampling strategy in the application of RGE to CNV detection.

### Applying RGE to speed up CNV detection

In this section, we demonstrate an example usage of RGE to speed up GENSENG, a GLM+NB based CNV detection method from read-count data of germline samples. GENSENG implements an HMM method. The underlying copy number is the hidden state variable, which emits probabilistic observations (i.e., the windowed read-count data). The main feature and advantage of GENSENG [10] is its ability simultaneously to segment read-count data and to correct the effect of confounders by fitting a NB regression in the HMM emission probability [10]. The NB regression model has the windowed read-counts as the response variable, copy number as the independent variable, and known confounders GC-content and mappability as covariates. GC-content is computed as the proportion of G or C bases in each window in the reference genome; and mappability is computed as the proportion of bases that can be uniquely aligned to the reference given a specific read length. Given the HMM setup, GENSENG applies the Baum-Welch algorithm [36] to estimate iteratively the most likely copy number for each window. In the Estimation step, it calculates the emission probability from the regression coefficients estimated in the previous round, while in the Maximization step it runs IRLS to estimate NB regression coefficients. RGE is implemented in the Maximization step such that only the sampled data of much reduced scale will be passed on to IRLS for estimating the NB regression coefficients. After each round of the Estimation-Maximization (E-M) iteration, the Baum-Welch algorithm generates the posterior probability of a window belonging to different copy numbers for each window. The iterations end when the algorithm converges. The GENSENG framework then assigns the copy number with the largest posterior probability to each window as the most likely copy number.

Algorithm 1 details R-GENSENG - the integration of RGE with GENSENG. In the equations below, **y** is the response variables vector (i.e., the read-counts in each window); **X** is the design matrix (i.e., copy number and covariate values in each window); $\mathbf {A} \in \mathbb {R}^{n \times m}$ is the posterior probabilities matrix with *n* windowns and *m* states. *a*_*ij*_ is the posterior probability that the *i*-th window belonging to the *j*-th state; *q*∈[0,1] is the proportion of the sample size to the entire size. RGE samples the rows using a weighted approach by assigning a sampling probability *h*∈[0,1] to the *i*-th window if it is a copy number variation window according to *p*_*i*_; otherwise RGE assigns 1−*h* to it as the sampling probability. To illustrate RGE in this study, we used a heuristic technique to choose a fixed value of *h*=0.99 or a downsampling rate of 1%, which is inspired by the CNV domain knowledge that less than 1% of windows have CNV. In real-life applications, the downsampling rate could be considered as a parameter for optimization, where runtime and sensitivity of RGE can be evaluated at a series of values of *h* and an optimal choice can then be made based on users specific needs on the runtime and sensitivity trade-off. Note that the weights are the posterior probabilities, which are available in each round of HMM inference, so there is no extra cost to obtain the weights. After sampling the reduced size data *X*^′^ and *y*^′^, an IRLS algorithm is applied to estimate the NB regression coefficients $\hat {\boldsymbol {\beta }}$ from *X*^′^ and *y*^′^ as an approximation of coefficients estimated from **X** and **y**. $\hat {\boldsymbol {\beta }}$ will be used in the next round Estimation step in GENSENG.



## Results and discussion

We conducted simulation and real data analyses to validate the statistical properties of RGE and to evaluate R-GENSENG’s performance (compared with GENSENG) for CNV detection.

### Validation of RGE’s statistical properties

We studied two properties of RGE. In the consistency study, we claim that the regression coefficients estimated by RGE will converge asymptotically at their true values. In the variance study, we claim that the weighted sampling used in our RGE yields a smaller estimated variance than that obtained using uniform sampling. In this section, we describe the empirical validation of these two properties using simulation.

We first simulated a series of read count data, each of which follows the NB distribution and is affected by the copy number variable and the covariates as described in the following NB regression model.

4 $$\begin{array}{@{}rcl@{}} \log(\mu) &=& \beta_{0} + \beta_{1} \log(CN) + \beta_{2}\log(l) + \beta_{3}\log(gc) \end{array} $$

where *μ* is the mean value of the read count data, *CN* is the copy number, *l* is the mappability score, *gc* is the GC content and the link function is the log link function [10]. We first generated the design matrix where each row represents a window and each of its three columns represents corresponding values for *l*, *gc*, and *CN*. To generate the covariate values, we used the chromosome 1 of the human reference genome (NCBI37) as the template and calculated the GC content and mappability in 10^6^ non-overlapping windows of 200bp in size (see Additional file 1). To generate the copy number values, we randomly selected 1% of the windows to be deletions (copy number 0 or 1) or duplications (copy number 3 to 6) and assigned the remaining 99% of windows to have copy number 2 (i.e., copy number neutral). We set the values of the coefficients *β*_1_,*β*_2_,*β*_3_ as 1,1 and 0.55 based on our experience. We then passed the design matrix (10^6^ rows and 3 columns) and the coefficients to the garsim function from R/gsarima to simulate read-count data with the mean of the NB regression following Eq. 4.

We next applied RGE to the simulated read-count data using two sampling proportions: 10% and 50%. Given each sampling proportion, we ran RGE 200 times. In each run, RGE sampled a subset of the data and returned coefficient estimates using the sampled data. By studying the distribution of the coefficient estimates from 200 replication runs, we can evaluate the convergence and the variance properties of RGE. To demonstrate the improvements RGE furnishes, we compared the coefficient estimates obtained by RGE to those by several alternative strategies: 1) the ground truth coefficients <1,1,0.55>; 2) the coefficients estimated using the entire dataset; and, 3) the coefficients estimated using a uniformly sampled subset of the data.

**Fig. 1:**
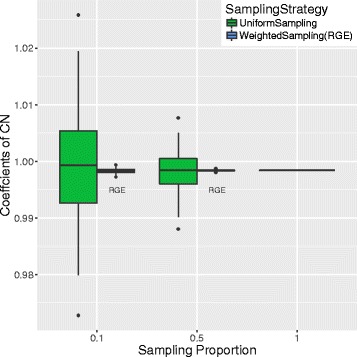
Simulation results for evaluating the RGE coefficient estimates on *CN*. The x-axis: sampling proportion; the y-axis: *CN* coefficient estimates. The ground truth is 1 at the y-axis. Boxplots are used to summarize the distributions of the coefficient estimates from 200 replication runs for each sampling strategy. The blue bars represent RGE (weighted sampling) given the sampling proportions (x-axis) 0.1 and 0.5. The green bars represent RGE (uniform sampling) given the sampling proportion (x-axis) 0.1 and 0.5. The segment at the x-axis-value of 1 represents the coefficient estimates using the entire dataset

The results from our simulation study are summarized in Fig. 1. We observe that 1) the RGE estimates converge at the ground truth, and 2) RGE yields a smaller estimated variance than does the uniform sampling subset. These results strongly support our claim that RGE is a consistent estimator with the desired variance property. Note that although the simulation experiments above were in CNV detection background, the conclusions are applicable in the more general GLM+NB based read-count analyses.

### R-GENSENG performance evaluation

Given the consistency and variance properties of RGE, we expect that R-GENSENG would be much faster than GENSENG while maintaining GENSENG’s accuracy in CNV calling. We carried out analyses on simulated and real data to evaluate empirically R-GENSENG’s performance.

#### Simulation study

The simulation study mimics a real-world scenario where we aim to detect CNVs from paired-end sequencing data generated from a CNV-containing chromosome. First, we created an artificial CNV-containing chromosome by implanting 200 CNVs into the chromosome 1 of the human reference genome (NCBI37). An implanted CNV is specified by its starting position (start_pos), ending position (end_pos) and type (duplication or deletion). To implant a duplication, we copied the base pairs within the affected region (start_pos to end_pos) immediately next to the affected region to create a tandem duplication. To implant a deletion, we removed the base pairs in the affected region similarly. Among the 200 CNVs, there were 119 deletions and 81 duplications. Among the implanted CNVs, there were 20 small CNVs (<1kbs), 86 median-size CNVs (between 1k and 3k bps), and 94 large CNVs (>3kbs). Next, we used the artificial chromosome as a template and applied wgsim, a sequencing simulator (part of the SAMTools) [37], to generate 100bps paired-end reads from the template. A total of 50 million paired-end reads were simulated yielding a sequencing coverage of 40x. The simulated reads were then aligned to the original chromosome 1 (NCBI37) to obtain the.bam file. Next, we divided the original chromosome 1 (NCBI37) into non-overlapping windows and computed read-count in each window. We chose four window sizes (i.e., 100bps, 200bps, 500bps, and 1000bps) to generate four sets of read-count data. Finally, we applied both GENSENG and R-GENSENG to each of the four read-count datasets. For R-GENSENG, we choose 0.99 for the sampling parameter *h* based on the fact that less than 1% of windows have CNV.

Using the implanted CNVs as the ground truth, we calibrated the sensitivity and false discovery rate (FDR) of R-GENSENG in comparison to GENSENG. Following [10], a true discovery is a reported CNV that satisfies two conditions: 1) having ≥50*%* reciprocal overlap with the ground truth CNV, and 2) having the same type (deletion or duplication) as the ground truth CNV. The sensitivity is calculated as the total number of true discoveries divided by the total number of ground truth CNVs. Similarly, a false discovery is a reported CNV that satisfies two conditions: 1) having <50*%* reciprocal overlap with a ground truth CNV, and 2) having the same type (deletion or duplication) as the ground truth CNV. The false discovery rate is calculated as the total number of false discoveries divided by the total number of reported CNVs. We compared the sensitivities and FDRs between GENSENG and R-GENSENG. The results are summarized in Tables 1 and 2.

**Table 1:** Sensitivity comparison between GENSENG and R-GENSENG

Window	Methods comparison (*G:*GENSENG,*R:*R-GENSENG)					
	Total CNV		Deletion		Duplication	
Size	*G*	*R*	*G*	*R*	*G*	*R*
100bps	188/200	187/200	112/119	112/119	76/81	75/81
	94%	94%	94%	94%	94%	93%
200bps	187/200	183/200	111/119	111/119	76/81	72/81
	94%	92%	93%	93%	94%	89%
500bps	169/200	168/200	99/119	99/119	70/81	69/81
	85%	84%	83%	83%	86%	85%
1000bps	125/200	121/200	78/119	75/119	47/81	46/81
	63%	61%	66%	63%	58%	57%

**Table 2:** FDR comparison between GENSENG and R-GENSENG

Window	Methods comparison ((*G:*GENSENG,*R:*R-GENSENG))					
	Total CNV		Deletion		Duplication	
Size	*G*	*R*	*G*	*R*	*G*	*R*
100bps	18/206	28/215	10/122	16/128	8/84	12/87
	8.7%	13.0%	8.2%	12.5%	9.5%	13.8%
200bps	10/197	14/197	3/114	5/116	7/83	9/81
	5.1%	7.1%	2.6%	4.3%	8.4%	11.1%
500bps	5/174	7/175	0/99	0/99	5/75	7/76
	2.9%	4%	0%	0%	6.7%	9.2%
1000bps	0/125	4/125	0/78	0/75	0/47	4/50
	0%	3.2%	0%	0%	0%	8%

In summary, the sensitivities of R-GENSENG are lower than that of GENSENG in all situations (i.e., different window sizes or different CNV types), but the differences in their sensitivities are small (<5*%* in all situations). These results suggest that R-GENSENG has comparable sensitivity with GENSENG. For read-count-based methods, the size of the windows is a tuning parameter [38]. Typically, as the window size gets larger relative to the size of the CNVs, it becomes more difficult to detect the CNVs. Our simulation results show that, when window size <1000bps, the sensitivities of both GENSENG and R-GENSENG were greater than 80%, whereas when window size was equals to 1000bps, it was hard to detect the small to median size CNVs, resulting in reduced sensitivities (<65%).

The FDRs of R-GENSENG are higher than the FDRs of GENSENG in all situations (i.e., different window size or different CNV type), but the differences in their FDRs are also small (<4.3*%* in all situations). These results suggest that R-GENSENG has a comparable FDR with GENSENG. In most of the situations (when window size >100bps), the FDRs of both GENSENG and R-GENSENG are small (<10*%*). When the window size is small (<100bps), both GENSENG and R-GENSENG have a relative higher FDR (>10*%*), presumably because it is more difficult to distinguish noise from true signal, especially for small CNVs.

In summary, our simulation study concluded that R-GENSENG has performance comparable to GENSENG in terms of sensitivity and FDR, and that both R-GENSENG and GENSENG are high in sensitivity and low in FDR.

#### Real data analyses

To further evaluate the relative performance of R-GENSENG, we applied R-GENSENG and GENSENG to the whole-genome sequencing data from three HapMap individuals sequenced as part of the 1000 Genomes Project [34, 35] (1000GP FTP sites: https://ftp.ncbi.nlm.nih.gov/1000genomes/ftp/pilot/_data/data/). Specifically, the CEU parent-offspring trio of European ancestry (NA12878, NA12891, NA12892), were sequenced to 40X coverage on average using the Illumina Genome Analyzer (I and II) platform. Sequencing reads were a mixture of single-end and paired-end with variable lengths (36bp, 51bp) and were aligned to the human reference genome NCBI37. The complete genome sequence data were obtained in the form of.bam alignment files from the 1000 GP FTP sites.

We focused on analyzing the 22 autosomes. Read quality control and input data preparation was done as previously described [10] (see Additional file 1). For each individual genome, we computed four sets of input data based on a varying window size of 100bps, 200bps, 500bps, and 1000bps.

**Fig. 2:**
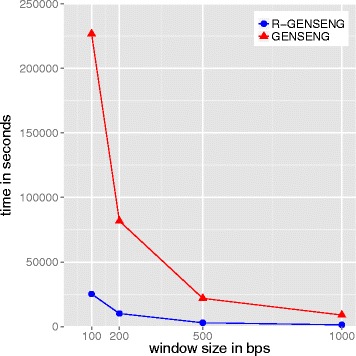
Running time of the real data with different window sizes. The x-axis is the window size and the y-axis is the running time (in seconds). The red curve connects the points representing the average running time of GENSENG at varying window sizes and the blue curve connects the points representing the average running time of R-GENSENG

First, we evaluated the running time of R-GENSENG compared to GENSENG, using four different window sizes (100bps, 200bps, 500bps and 1000bps) and corresponding numbers of windows 25 million, 12.5 million, 5 million, and 2.5 million. The running time includes the time to read the input, the inference time, and the the time to write output to disk. The time to generate the read count data, which is the same between R-GENSENG and GENSENG, is excluded. We recorded the running time on inference in seconds for each sample and averaged the running time among the three samples. We compared the average running time between GENSENG and R-GENSENG across varying window sizes in Fig. 2. From Fig. 2 we find that: 1) R-GENSENG is nearly one order of magnitude faster than GENSENG across all window sizes; and, 2) when the window size is small (100bps) and the scale of the data is huge (25 million windows), the reduction in running time with AS-GENSENG is remarkable (i.e., R-GENSENG uses 6 hours but GENSENG uses 60 hours).

Next we evaluated the relative accuracy of R-GENSENG for CNV calling. We had evaluated previously the accuracy of GENSENG using the same data [34, 35] and compared GENSENG to the best performing read-count-based method CNVnator [8]. We found that GENSENG had a sensitivity of 50% averaged over the three samples, which is better than CNVnator (10% higher sensitivity and comparable specificity) [10]. In this study, we use the CNV calls from GENSENG as the benchmark data, intersected the CNV calls from R-GENSENG with that of GENSENG (using a 50% reciprocal overlapping condition), and reported the proportions of GENSENG calls overlapped by R-GENSENG. The results are summarized in Table 3. Given the consistency and variance properties demonstrated in the previous Sections, we expected that R-GENSENG would be highly concordant with GENSENG calls. From Table 3, we found that the overlapping proportions are >0.92 for most cases, which is acceptable when speed is a concern. The only scenario when the discrepancy can be high (18%) is when the window size is 100bp. However, modern day sequencing technologies use reads that are more than100bp and therefore a window-size of 100bp will never be used in practice (window size must be at least 2 times of the read length).

**Table 3:** The proportions of GENSENG calls overlapped by R-GENSENG calls

Window Size	NA12878	NA12891	NA12892
100bps	0.95	0.84	0.82
200bps	0.92	0.95	0.93
500bps	0.98	0.98	0.97
1000bps	0.97	0.97	0.97

In summary, R-GENSENG runs much faster than GENSENG while preserving the accuracy of GENSENG in CNV calling.

## Conclusions

A variety of genomic assays have adopted the HTS technologies to quantify the amount of molecules or enriched genome regions in the form of read-count data. However, while the GLM+NB based methods provide a statistically powerful tool to discover the true relationship between biological factors from the read count data, the computational bottleneck of the GLM+NB methods hinders their application to large-scale genomic data. In this study, we have proposed an efficient regression coefficients estimator, RGE, to accelerate substantially the estimation procedure. Based on a randomized algorithm, RGE selects a subset of data with remarkably reduced size and estimates the regression coefficients based on the data subset. We have shown both theoretically and empirically that RGE is statistically consistent and yields a low variance. As a demonstration of the application of RGE to existing GLM+NB methods, we also introduced the algorithm to embed RGE in the read-count based CNV detection framework GENSENG [10]. The resulting R-GENSENG method not only runs much faster than GENSENG but also keeps GENSENG’s CNV calling accuracy, based on both simulation and empirical studies. Comparing R-GENSENG with GENSENG, R-GENSENG is almost identical to GENSENG except for applying the RGE to estimate the sub-optimal regression coefficients estimator in each round of the iteration. As we have demonstrated, R-GENSENG is much faster than GENSENG but has a slight deficiency in terms of the accuracy. For applications using large-scale windowed read count data, such as whole-genome CNV detection with DNA-seq data, peak detection with ChIP-seq data and genome-wide epigenetic studies, we recommend using the randomized approach when the speed/computation cost is a concern. The randomized approach is not appropriate for RNA-seq data analysis, where reads are counted using a gene as the counting unit and differential analysis is done gene by gene [14, 15, 39–43].

## Additional file

Additional file 1 Proof of Theorem 1 and descriptions of the GLM+NB HMM model. (PDF 301 kb)

## Abbreviations

CNV: Copy-number variants
GLM: Generalized linear models
HTS: High-throughput sequencing
NB: Negative-binomial
RGE: Randomized GLM+NB coefficients estimator
