# Supplementary material for: A randomized approach to speed up the analysis of large-scale read-count data in the application of CNV detection
Source: BMC Bioinformatics. 2018 Mar 1;19:74. doi: 10.1186/s12859-018-2077-6 (PMC5831535; doi:10.1186/s12859-018-2077-6)
Supplement: Supplementary file 1 — Proof of Theorem 1 and descriptions of the GLM+NB HMM model. (PDF 301 kb) [file 12859_2018_2077_MOESM1_ESM.pdf]

A RANDOMIZED APPROACH TO SPEED UP THE ANALYSIS OF LARGE-SCALE READ-COUNT DATA IN THE APPLICATION OF CNV DETECTION

WEIBO WANG, WEI SUN, WEI WANG AND JIN SZATKIEWICZ

1. The Proof of Consistency of RGE

1.1. Using IRLS to estimate GLM-NB model parameters. We first demonstrate using IRLS to estimate GLM-NB model parameters.

The log likelihood for a negative binomial model is

$$l(\mathbf{y};\boldsymbol{\beta},\phi) = \sum_{i=1}^{n}\left[\log\left(\frac{\Gamma(y_i+1/\phi)}{y_i!\Gamma(1/\phi)}\right) + y_i\log\left(\frac{\phi\mu_i}{1+\phi\mu_i}\right) - \frac{1}{\phi}\log(1+\phi\mu_i)\right], \tag{1}$$

where $\mathbf{y}$ is the response vector of length n, $\boldsymbol{\beta}$ is the coefficients vector of length p, ϕ is the negative binomial over-dispersion parameter, and $\mu_i = E(y_i)$. Consider the generic form of a GLM model

$$l(\mathbf{y};\boldsymbol{\beta},\varphi) = \sum_{i=1}^{n} l_i = \sum_{i=1}^{n}\left\{\varphi^{-1}[y_i\theta_i - b(\theta_i)] + c(y_i,\varphi)\right\}$$

where $\mathbf{y}$ is the response vector of length n, $\boldsymbol{\beta}$ is the coefficients vector of length p, φ is the GLM dispersion parameter. Assuming the over-dispersion parameter ϕ is fixed, then a negative binomial distribution belongs to the exponential family. Thus matching it with the generic form of a GLM model, we have

$$\begin{aligned}
\varphi &= 1 \\
\theta_i &= \log\left(\frac{\phi\mu_i}{1+\phi\mu_i}\right), \quad \frac{\partial\theta_i}{\partial\mu_i} = \frac{1}{V(\mu_i)} = \frac{1}{\mu_i+\phi\mu_i^2} \\
b(\theta_i) &= \frac{1}{\phi}\log(1+\phi\mu_i) = -\frac{1}{\phi}\log[1-\exp(\theta_i)], \quad b'(\theta_i) = \mu_i, \quad b''(\theta_i) = V_i \\
c(y_i,\varphi) &= \frac{\Gamma(y_i+1/\phi)}{y_i!\Gamma(1/\phi)}
\end{aligned}$$

Let $\eta_i = x_i^T\boldsymbol{\beta} = g(\mu_i)$, where g is a link function. In our model, $\eta_i = g(\mu_i) = \log(\mu_i)$.

To derive the the MLE of β_j, we start with the score function and Fisher's information matrix. The score function is

$$S_j = \frac{\partial l(\mathbf{y};\boldsymbol{\beta},\varphi)}{\partial\beta_j} = \sum_{i=1}^{n}\frac{\partial l_i}{\partial\theta_i}\frac{\partial\theta_i}{\partial\mu_i}\frac{\partial\mu_i}{\partial\eta_i}\frac{\partial\eta_i}{\partial\beta_j} = \sum_{i=1}^{n}(y_i-\mu_i)\frac{1}{V(\mu_i)}\frac{1}{g'(\mu_i)}x_{ij}. \tag{2}$$

Let I_{jk} be the (j,k)-th element of the Fisher's information matrix,

$$I_{jk} = \sum_{i=1}^{n} E\left[\frac{\partial l_i}{\beta_j}\frac{\partial l_i}{\beta_k}\right] = \sum_{i=1}^{n} E\left\{\frac{(y_i-\mu_i)^2}{[V(\mu_i)g'(\mu_i)]^2}x_{ij}x_{ik}\right\} = \sum_{i=1}^{n}\left\{\frac{1}{V(\mu_i)\,[g'(\mu_i)]^2}x_{ij}x_{ik}\right\},$$

and the last equation is due to the fact that $E[(y_i-\mu_i)^2] = V(\mu_i)$.

Let $I^{(t-1)} = I(\boldsymbol{\beta})|_{\boldsymbol{\beta}=\boldsymbol{\beta}^{(t-1)}}$ and $S^{(t-1)} = \partial l/\partial\boldsymbol{\beta}|_{\boldsymbol{\beta}=\boldsymbol{\beta}^{(t-1)}}$. By Fisher scoring, the update of $\boldsymbol{\beta}$ from the $(t-1)$-th iteration to the t-th iteration is

$$\boldsymbol{\beta}^{(t)} = \boldsymbol{\beta}^{(t-1)} + \left[I^{(t-1)}\right]^{-1} S^{(t-1)} \Rightarrow I^{(t-1)}\boldsymbol{\beta}^{(t)} = I^{(t-1)}\boldsymbol{\beta}^{(t-1)} + S^{(t-1)}.$$

Let W be a diagonal $n\times n$ matrix, with the i-th diagonal element $w_i = 1/\{V(\mu_i)\,[g'(\mu_i)]^2\}$ for $i=1, ..., n$. Then based on equations (2) and (3), the score function and information matrix can be written as

$$S = \mathbf{X}W\boldsymbol{\zeta} \;\text{ and }\; I = \mathbf{X}^TW\mathbf{X},$$

where $\mathbf{X}$ is the design matrix, $\boldsymbol{\zeta}$ is a vector of length n and $\zeta_i = (y_i-\mu_i)g'(\mu_i)$. When W is evaluate based on $\boldsymbol{\beta}^{(t-1)}$, we write it as $W^{(t-1)}$. Then the Fisher scoring equation can be written as

$$[\mathbf{X}^TW^{(t-1)}\mathbf{X}]\boldsymbol{\beta}^{(t)} = \mathbf{X}^TW^{(t-1)}\mathbf{X}\boldsymbol{\beta}^{(t-1)} + \mathbf{X}W^{(t-1)}\boldsymbol{\zeta} = \mathbf{X}^TW^{(t-1)}\left[\boldsymbol{\eta}^{(t-1)}+\boldsymbol{\zeta}\right].$$

Therefore, $\boldsymbol{\beta}^{(t)}$ is the solution of weighted least squares with working response being $\mathbf{z} = \boldsymbol{\eta}+\boldsymbol{\zeta}$, and $z_i = x_i\boldsymbol{\beta} + (y_i-\mu_i)g'(\mu_i)$, and weight for the i-th observation is $1/\{V(\mu_i)\,[g'(\mu_i)]^2\}$. Here we use log link function $g(\mu_i) = \log(\mu_i)$, and thus

$$z_i = x_i\boldsymbol{\beta} + (y_i-\mu_i)/\mu_i, \;\text{ and } w_i = \frac{\mu_i^2}{\mu_i+\mu_i^2\phi} = \frac{\mu_i}{1+\mu_i\phi}.$$

1.2. **Proof of Theorem 1.** Here we provide proof details of Theorem 1. For equation

$$f(\boldsymbol{\beta}) = \mathbf{X^T}(\mathbf{m}\circ\mathbf{X}\boldsymbol{\beta}) - \mathbf{X^T}(\mathbf{m}\circ\mathbf{y}), \tag{3}$$

where $\mathbf{X}\in\mathbb{R}^{n\times p}$ is the original design matrix times the square root of weight matrix W, n is the number of rows, p is the number of columns, $\mathbf{y}\in\mathbb{R}^n$ is a n-dimensional known vector. Let m_i be the sampling indicator for the i-th entry, $i=1,...,n$; $m_i=1$ means that the i-th entry is sampled, $m_i=0$ means otherwise. We denote $\|\mathbf{X}\|_\infty$ the L_∞ norm of a matrix $\mathbf{X}$, which is the maximum absolute row sum of the matrix. We denote $\|\mathbf{v}\|_\infty$ the L_∞ norm of a vector $\mathbf{v}$, which is the maximum absolute value of the elements.

Theorem 1 states that there exists a solution when Equation 3 equals 0 that is inside the hypercube of the true coefficients $\boldsymbol{\beta}_0$.

Let

$$\begin{aligned} f(\boldsymbol{\delta}) &= \mathbf{X^T}(\mathbf{m}\circ\mathbf{X}\boldsymbol{\delta}) - \mathbf{X^T}(\mathbf{m}\circ\mathbf{y}) \\ &= \mathbf{X^T}(\mathbf{m}\circ\mathbf{X}\boldsymbol{\delta}) - \mathbf{X^T}(\mathbf{m}\circ\mathbf{X}\boldsymbol{\beta_0}) - \left[\mathbf{X^T}(\mathbf{m}\circ\mathbf{y}) - \mathbf{X^T}(\mathbf{m}\circ\mathbf{X}\boldsymbol{\beta_0})\right] \\ &= \mathbf{X^T}(\mathbf{m}\circ\mathbf{X}\boldsymbol{\delta}) - \mathbf{X^T}(\mathbf{m}\circ\mathbf{X}\boldsymbol{\beta_0}) - \mathbf{X^T}\left[\mathbf{m}\circ(\mathbf{y}-\mathbf{X}\boldsymbol{\beta_0})\right] \end{aligned}$$

Thus proving Theorem 1 is equivalent to prove that there exists a solution inside the hypercube $\mathcal{N}$ to satisfy $f(\boldsymbol{\delta}) = 0$.

After expanding $\mathbf{X^T}\left(\mathbf{m}\circ\mathbf{X}\boldsymbol{\delta}\right)$ around $\boldsymbol{\beta}_0$ by the second order Taylor expansion and inserting it into $f(\boldsymbol{\delta})$ we have,

$$f(\boldsymbol{\delta}) = \left[\mathbf{X^T}\mathrm{diag}\left(\mathbf{m}\right)\mathbf{X}\right](\boldsymbol{\delta}-\boldsymbol{\beta}_0) - \boldsymbol{\xi} + \mathbf{r},$$

where $\boldsymbol{\xi} = (\xi_1, ..., \xi_p)^T = \mathbf{X^T}\left[\mathbf{m}\circ(\mathbf{y}-\mathbf{X}\boldsymbol{\beta_0})\right] = \mathbf{X^T}\boldsymbol{\epsilon}$, $\mathbf{r} = (r_1, ..., r_p)^T$ are Lagrange reminders and for each $j = 1, ..., p$,

$$r_j = \frac{1}{2}(\boldsymbol{\delta}-\boldsymbol{\beta}_0)\nabla^2\left[\mathbf{x_j^T}(\mathbf{m}\circ\mathbf{X}\boldsymbol{\delta})\right](\boldsymbol{\delta}-\boldsymbol{\beta}_0),$$

It is straightforward to see the second derivative $\nabla^2\left[\mathbf{x_j^T}(\mathbf{m}\circ\mathbf{X}\boldsymbol{\delta})\right] = 0$, so we have $\|\mathbf{r}\|_\infty = 0$,

Let

$$\bar{f}(\boldsymbol{\delta}) = \left[\mathbf{X^T}\mathrm{diag}\left(\mathbf{m}\right)\mathbf{X}\right]^{-1} f(\boldsymbol{\delta}) = \boldsymbol{\delta}-\boldsymbol{\beta}_0+\mathbf{u},$$

where $\mathbf{u} = -\left[\mathbf{X^T}\mathrm{diag}\left(\mathbf{m}\right)\mathbf{X}\right]^{-1}[\boldsymbol{\xi}-\boldsymbol{r}]$.

The range $\|\mathbf{u}\|_\infty$, is determined by $\|\left[\mathbf{X^T}\mathrm{diag}\left(\mathbf{m}\right)\mathbf{X}\right]^{-1}\|_\infty$ and $\|\boldsymbol{\xi}\|_\infty$ (we already know that $\|\mathbf{r}\|_\infty = 0$).

Condition 1.1. $\|\left[\mathbf{X^T}\mathit{diag}\left(\mathbf{m}\right)\mathbf{X}\right]^{-1}\|_\infty = O(n^{-1})$.

Condition 1.1 ensures that the sampled matrix $\mathbf{X^T}\mathrm{diag}\left(\mathbf{m}\right)\mathbf{X}$ is not singular. The CNV problem setting (where copy number and intercept are set as two covariates) could be used as an illustration example to explain Condition 1.1 is reasonable. Without the loss the generality we assume the j-th column ($j = 1, 2$) of the sampled data has been standardized such that $\overline{\mathbf{m}\circ\mathbf{x_j}} = 0$, and $\|\mathbf{m}\circ\mathbf{x_j}\|_2 = \sqrt{n_0}$, where n_0 is the size of the sampled data. If the copy number and the intercept have not been standardized, the conclusion still holds with $\|\mathbf{m}\circ\mathbf{x_j}\|_2$ assumed to be in the order of $\sqrt{n_0}$. $\left[\mathbf{X^T}\mathrm{diag}\left(\mathbf{m}\right)\mathbf{X}\right]$ becomes $\mathrm{diag}\left(n_0, n_0\right)$. The inverse is $\mathrm{diag}\left(n_0^{-1}, n_0^{-1}\right)$ and the L_∞ norm is $n_0^{-1} = O(n^{-1})$.

We study $\|\boldsymbol{\xi}\|_\infty$ from probability perspective. We first define the event $\mathcal{E} = \left\{\|\boldsymbol{\xi}\|_\infty \le c^{-1/2}\sqrt{n_0\log n_0}\right\}$, where $\sqrt{n_0}$ is the L_2 norm of vector $\mathbf{m}\circ\mathbf{x_j}$. [5] proves the following proposition,

Proposition 1.2. $P\left(|\mathbf{a^T}\mathbf{Y} - \mathbf{a^T}\mathbf{b}'\left(\theta_\mathbf{0}\right)| > \|\mathbf{a}\|_2\varepsilon\right) \le \psi\left(\varepsilon\right)$,

where $\mathbf{a}\in\mathbb{R}^n$, $\varepsilon\in(0, \|\mathbf{a}\|_2/\|\mathbf{a}\|_\infty]$, $c = 1/(2v_0+2M)$ for some $M, v_0 \in (0,\infty)$ such that Y, M, v_0, and $b'\left(\theta_0\right)$ satisfy the moment condition (20) in [5], and $\psi\left(\varepsilon\right) = 2\exp^{-c\varepsilon^2}$. Using the proposition, the probability that event $\mathcal{E}$ happens could be calculated as

$$\begin{aligned} P\left(\mathcal{E}\right) &\ge 1 - \sum_{j=1}^{p} P\left(|\boldsymbol{\xi}_j| \ge c^{-1/2}\sqrt{n_0\log n_0}\right) \\ &\ge 1 - 2[pn_0^{-1}], \end{aligned}$$

where $\mathbf{a}$ corresponds to $\mathbf{m}\circ\mathbf{x_j}$, and $\varepsilon = c^{-1/2}\sqrt{\log n_0}$. The probability goes to 1 when n_0 goes to ∞. Thus the event $\mathcal{E}$ holds when n_0 goes to ∞. Thus $\|\boldsymbol{\xi}\|_\infty \le c^{-1/2}\sqrt{n_0\log n_0} = O(n^{1/2}\sqrt{\log n})$.

When Condition 1.1 is ensured, and given $\|\boldsymbol{\xi}\|_\infty = O(n^{1/2}\sqrt{\log n})$, we have

$$\begin{aligned} \|\mathbf{u}\|_\infty &\le \|\left[\mathbf{X^T}\mathrm{diag}\left(\mathbf{m}\right)\mathbf{X}\right]^{-1}\|_\infty(\|\boldsymbol{\xi}\|_\infty + \|\mathbf{r}\|_\infty) \\ &= O(n^{-1/2}\sqrt{\log n}), \end{aligned}$$

so $\|\mathbf{u}\|_\infty = o(n^{-\gamma_0}\sqrt{\log n})$ for some $\gamma_0\in(0,1/2)$. Since for any $\boldsymbol{\delta}\in\mathcal{N}$, we have $\|\boldsymbol{\delta}\|_\infty \ge \|\boldsymbol{\beta}_0\|_\infty - d_n$, where $d_n \equiv 2^{-1}\min_{1\le j\le p}\{|\beta_{0j}|\} = O(n^{-\gamma_0}(\log n)^{1/2})$ for some $\gamma_0\in(0,1/2)$. Therefore we have

$$\min_{j=1,\dots,p}\|\delta_j\| \ge \min_{j=1,\dots,p}\|\boldsymbol{\beta}_{0j}\| - d_n = d_n.$$

For a constant $C > 0$ and sufficiently large n, if $\delta_j - \beta_j = Cn^{-\gamma_0}\sqrt{\log n}$, $\bar{f}_j(\boldsymbol{\delta}) \geq Cn^{-\gamma_0}\sqrt{\log n} - \|\mathbf{u}\|_\infty \geq 0$. And if $\delta_j - \beta_j = -Cn^{-\gamma_0}\sqrt{\log n}$, $\bar{f}_j(\boldsymbol{\delta}) \leq -Cn^{-\gamma_0}\sqrt{\log n} + \|\mathbf{u}\|_\infty \leq 0$. Because the continuity of function $\bar{f}(\boldsymbol{\delta}) = (\bar{f}_1(\boldsymbol{\delta}), ..., \bar{f}_p(\boldsymbol{\delta}))$, and Miranda's existence theorem, there is a solution $\hat{\boldsymbol{\beta}}$ for $f(\boldsymbol{\delta}) = 0$ in $\mathcal{N}$, i.e., there is a solution for Equation 3 in $\mathcal{N}$. Thus Theorem 1 holds.

2. GENSENG CNV Detection Framework

GENSENG's analytic protocol comprises three steps: 1. Input data preparation (including read quality control and Computation of read-depth and covariate values); 2. HMM inference of copy number while correcting for biases; 3. Post-segmentation processing. We introduced each step in the following sections.

2.1. **Data Preparation.**

We first applied the following steps to control the quality of the raw sequencing reads. 1. Remove any read that fails platform/vendor quality checks, or either a PCR duplicate or an optical duplicate. 2. Extract all single-end reads and properly paired paired-end reads. 3. Extract confidently aligned reads with MAPQ $\geq$ a specified threshold. In this study, we use MAPQ $\geq$10, which was empirically determined.

We then divided the genome into consecutive windows. In selection of window size, we used a sliding window approach of 200bps non-overlapping windows. The size of the window was empirically determined.

Finally we obtained the read-depth in each window by counting the number of reads in each window. Each read (e.g. 36-mer or 51-mer from the 1000 Genomes Project data[1, 7]) is represented by its middle base pair. A fragment is counted where read mapping information is available.

(1) If two ends of a pair fall in two windows, assign 1/2 to each window where the ends fall;
(2) If both ends of a pair fall in the same window, assign 1 to the window;
(3) If paired-ended but only one-end present, assign 1/2 to the window where the ends fall;
(4) If single-end, always assign 1 to the window where the end falls.

Covariates were calculated as a quantitative measurement of bias at each window. In this study, the set of covariates include GC content and mappability score. GC content is computed as in the following steps. (1) Calculate the proportion of G or C bases in each window from a given reference genome.(2) Apply a cubic spline smoothing and then transform the GC proportion based on the fitted curve so that the transformed GC proportion and logarithm of the read-depth are linearly correlated. (3) The transformed GC proportion is median-centered and is referred to as GC content hereafter. Mappability score is computed as in the following steps. (1) Align the K-mers starting at each base position back to reference genome using a desired aligner, e.g. BWA [6]. (2) Identify base positions where the corresponding K-mers are correctly aligned (i.e. there is a unique best hit and it is the true position of the K-mer). (3) Compute mappability score as the proportion of correctly-aligned bases (a.k.a. mappable bases) in a given window.

In summary, the input data is a triplet for each window represent by $\{O, G, L\} = \{o_1......o_T, g_1......g_T, l_1.....l_T\}$, where T is the total number of windows of a chromosome, o_t denotes the read-depth, g_t denotes the GC content, and l_t denotes the mappability score of the tth window.

2.2. **HMM setup.**

We use a time-homogeneous discrete hidden Markov model (HMM) to segment the genome to regions of same copy number. In our HMM, time represents the sliding windows tiled along a chromosome, denoted by t.

- The state represents the underlying copy number (CN). The state variable $q_t = CN_t$ is hidden and discrete with N possible values, $(0, 1, ..., N-1)$, where N, is derived from the data. A particular sequence of the states is described by $q = (q_1, ..., q_T)$, where T is the total number of sliding windows of a chromosome. Let π_j be the initial state probability, the probability that the state of the first window is state j. The underlying hidden Markov chain is defined by state transitions $P(q_t|q_{t-1})$ and is represented by a time-independent stochastic transition matrix $A = \{a_{jz}\} = P(q_t = z|q_{t-1} = j)$.
- Each copy number state emits an observation, the read-depth. The observation variable, O_t, is a discrete count variable. A particular sequence of the observations is described by $o = (o_1, ..., o_T)$. The emission probability of a particular observation at a particular time t for state j is described by $e(t, j) = P(O_t = o_t|q_t = j)$. For a detailed description of the emission probability, see Section 2.3.
- We use the Baum-Welch algorithm [2] to find the maximum likelihood estimates (MLE) of the HMM parameters. Following Bilmes [3], we define the complete-date likelihood and solve the Q function in order to find the maximum likelihood estimates (MLE) of the HMM parameters.

2.3. **Emission probability.** The emission probability of the read-depth, $e(t, j) = P(O_t = o_t|q_t = j)$, is modeled as a mixture of a uniform distribution and a negative binomial distribution.

$$e(t, j) = c/R_m + (1 - c)e^{NB}(t, j) \tag{4}$$

where c is the proportion of the random uniform component and is fixed as constant for each state; and R_m is the largest read-depth among all windows and thus $1/R_m$ is the uniform density.

To describe the negative binomially distributed component, $e^{NB}(t, j)$, we first explain the relationship between the Poisson and the negative binomial distributions. The Poisson distribution imposes that the variance equals to the mean. The negative binomial distribution allows overdispersion. Specifically, if O follows a Poisson distribution with mean μ, and μ follows a gamma distribution, the resulting distribution for O is a negative binomial distribution. The variance of negative binomial distribution is $\mu_t + \phi\mu_t^2$, where $\phi\mu_t^2$ is the overdispersion part of the variance. As $\phi \to 0$, $f_{NB}(o_t; \mu_t, \phi)$ reduces to a Poisson distribution with mean μ_t and variance μ_t. $f_P(o_t; \mu_t) = \frac{\exp(-\mu_t)\mu_t^{o_t}}{o_t!}$.

Next, the mean value of the negative binomially distributed component is expressed as a function of a set of covariates to account for confounders.

$$\mu_{tj} \quad = \quad \alpha_0 * (CN_t)^{\beta_1} * (l_t)^{\beta_2} * (g_t)^{\beta_3} \tag{5}$$

where t denotes the tth window, j is the index of the copy number state, j emphasizes the dependency of the mean μ_t on the copy number CN_t, l_t is the mappability score, g_t is the GC content. For computational convenience, we set $CN_t = 0.5$ when $j = 0$, and set $CN_t = j$ when $j > 0$.

We then employ a log link function to acknowledge the fact that $\mu_{tj} > 0$ and obtain:

$$\log(\mu_{tj}) \quad = \quad \beta_0 + \beta_1 * \log(CN_t) + \beta_2 * \log(l_t) + \beta_3 * \log(g_t) \tag{6}$$

$\beta_0, \beta_1, \beta_2, \beta_3$ are the regression coefficients. Specifically, $\beta_0 = \log(\alpha_0)$, is the intercept parameter and is interpreted as the average level of read-depth signal when all covariates are equal to zero. β_1 is the amount of increase of read-depth for every unit increase of copy number, CN. β_2 is the amount of increase of read-depth for every unit increase of the mappability score, l. β_3 is the amount of increase of read-depth for every unit increase of the GC content, g.

Thus, given the above regression model for the mean, the negative binomial probability distribution function is expressed as the following:

$$e^{NB}(t,j) = f_{NB}(o_t;\mu_{tj},\phi_j) \quad = \quad \frac{\Gamma(o_t+1/\phi_j)}{o_t!\Gamma(1/\phi_j)}\left(\frac{1}{1+\phi_j\mu_{tj}}\right)^{1/\phi_j}\left(\frac{\phi_j\mu_{tj}}{1+\phi_j\mu_{tj}}\right)^{o_t} \tag{7}$$

The complete emission probability is then expressed as the following:

$$e(t,j) \quad = \quad c/R_m + (1-c)\frac{\Gamma(o_t+1/\phi_j)}{o_t!\Gamma(1/\phi_j)}\left(\frac{1}{1+\phi_j\mu_{tj}}\right)^{1/\phi_j}\left(\frac{\phi_j\mu_{tj}}{1+\phi_j\mu_{tj}}\right)^{o_t} \tag{8}$$

2.4. **The Program Flow of HMM inference.** A time-homogeneous HMM has been implemented in C++.

- HMM input: $\{\mathbf{O},\mathbf{G},\mathbf{L}\}$ and Λ_0. Here $\{\mathbf{O}\}$ is the read-depth, $\{\mathbf{G}\}$ is the GC content, and $\{\mathbf{L}\}$ is the mappability score computed for each sliding window. Λ_0 is either the initial values of the HMM parameters or the parameter estimates from the previous iteration. The HMM parameters include the state parameters and the emission parameters.
- HMM output: The estimated HMM parameters Λ_1. The log likelihood from each iteration, $\log(p(\mathbf{O}|\Lambda))$.
- A one-step update of the Baum-Welch algorithm [2] is illustrated below. The expectation (E-step) and the maximization (M-step) procedures iterate until the convergence criterion (smaller than 10^{-6} change in the log-likelihood) is reached.
- Most of the computations are carried out in log scale to avoid underflow or overflow. A utility function `logsumexp` is used to facilitate the computation. Specifically, it is defined as $\texttt{logsumexp}_j(v) = \log\left(\sum_j \exp(v_j)\right)$, where $v = \{v_j\}$ is a vector.
- For efficient implementation, we estimate the $\log(\mu_{tj})$ directly using the IRLS method. Alternative approach could be estimating the regression coefficients.

Below we give details about Model Initialization steps:

(a) The number of states:

N is found from the data. Here we assume N=7, for CN = 0,1,2,3,4,5,6+.

(b) Initial state probability, π_j:

For state CN = 2: 0.9995; for other states: (1-0.9995)/(N-1).

(c) Initial state transition probability, a_{jz}:

Self-transition probability: for state CN = 2: 0.9995; for other states: 0.995;

Transition probability to other states, i.e. a_{jz} when $z \neq j$:

Transiting from CN = 2 to $CN < 2$: (1-0.9995)/3; from CN = 2 to $CN > 2$: (1-0.9995)/12;

Transiting from $CN < 2$ to CN = 2: (1-0.995)/9; from $CN < 2$ to $CN < 2$: (1-0.995)/90; from $CN < 2$ to $CN > 2$: (1-0.995)/400;

Transiting from $CN > 2$ to $CN < 2$: (1-0.995)/40; from $CN > 2$ to CN = 2: (1-0.995)/1.25; from $CN > 2$ to $CN > 2$: (1-0.995)/20;

(d) Initial mean values of the negative binomially distributed component, $\log(\mu_{tj})$:

Assume normal copy number (CN=2) for all windows.

Set $\beta_3 = 0.5$. β_3 is the coefficient for GC content. 0.5 is the empirically determined value.

intercept=$\log(\text{median}(O)) - \log(2) - \text{median}(\log(L)) - \beta_3\text{median}(G)$.

for $j = 0$, offset $= \log(0.5) + \log(l_t)$.

for $j = 1..N-1$, offset $= \log(j) + \log(l_t)$.
$\log(\mu_{tj}) =$ intercept $+$ offset $+ \beta_3 g_t$.
(e) Initial overdispersion parameters, ϕ_j:
In this study, we have one overdispersion parameter ϕ for different states jointly through setting $\phi_j = \phi$. And set $\phi = 1$ for initialization.

(f) Initial mixing probability, c:
$c = 0.01$. The mixing probability is the same for the normal state and the other states.

(g) Initial parameter for the uniform distribution, R_m:
R_m=max($\mathbf{O}$).
The details about the E-step of the EM procedures were as follows:
Given the current parameter estimates Λ_0, we efficiently compute the desired quantities.

2.4.1. *The Emission Probability.*

$$e(t,j) = c/R_m + (1-c)\frac{\Gamma(o_t + 1/\phi_j)}{o_t!\Gamma(1/\phi_j)}\left(\frac{1}{1+\phi_j\mu_{tj}}\right)^{1/\phi_z}\left(\frac{\phi_j\mu_{tj}}{1+\phi_j\mu_{tj}}\right)^{o_t} \quad (9)$$

2.4.2. *The Forward Probability.*

$$f(t,j) = P(o_1, o_2, ...o_t, q_t = j \texttt{ ends at } t|\Lambda_0) \quad (10)$$

Algorithm

(1) Initialization:

$$f(1,j) = \pi_z e(1,j) \quad (11)$$

$$\log(f(1,j)) = \log(\pi_j) + \log(e(1,j)) \quad (12)$$

(2) Recursion, for $t \in (2:T)$ and for $j \in (1:N)$,

$$f(t,j) = e(t,j)\sum_j f(t-1,j)a_t(z,j) \quad (13)$$

$$\log(f(t,j)) = \log(e(t,j)) + \texttt{logsumexp}_j\left[\log(f(t-1,j)) + \log(a_t(j,z))\right] \quad (14)$$

(3) Termination: computation of the overall likelihood $\log(p(\mathbf{O}|\Lambda_0))$

$$p(\mathbf{O}|\Lambda_0) = \sum_z f(T,z) \quad (15)$$

$$\log(p(\mathbf{O}|\Lambda_0)) = \texttt{logsumexp}_z \log(f(T,z)) \quad (16)$$

2.4.3. *The Backward Probability.*

$$b(t,z) = P(o_{t+1}, o_{t+2}, ...o_T|q_t = z \texttt{ ends at } t|\Lambda_0) \quad (17)$$

Algorithm

(1) Initialization:

$$b(T,z) = 1 \quad (18)$$

$$\log(b(T,z)) = 0 \quad (19)$$

(2) Recursion, for $t \in (T:2)$ and for $z \in (1:N)$,

$$b(t-1,z) = \sum_j \left[a_t(z,j)e(t,j)b(t,j)\right] \tag{20}$$

$$\log(b(t-1,z)) = \texttt{logsumexp}\left[\log(a_t(z,j)) + \log(e(t,j)) + \log(b(t,j))\right] \tag{21}$$

2.4.4. *The Posterior Probability.*

$$\gamma(t,j) = P(q_t = j|\mathbf{O}, \Lambda_0) \tag{22}$$

Algorithm

$$\gamma(t,j) = \frac{f(t,j)b(t,j)}{p(\mathbf{O}|\Lambda_0)} \tag{23}$$

$$\log(\gamma(t,j)) = \log(f(t,j)) + \log(b(t,j)) - \log(p(\mathbf{O}|\Lambda_0)) \tag{24}$$

The details about the M-step of the EM procedure were given below:

2.4.5. *Estimate the initial state probability π_j.* The initial probability π_j is simply the posterior probability of being state j at position 1, therefore the new estimate of π_j, denoted by $\overline{\pi}_j$, is computed as the following:

$$\overline{\pi}_j = \frac{f(1,j)b(1,j)}{p(\mathbf{O}|\Lambda_0)}, \tag{25}$$

$$\log(\overline{\pi}_j) = \log(f(1,j)) + \log(b(1,j)) - \log(p(\mathbf{O}|\Lambda_0)). \tag{26}$$

2.4.6. *Estimate the transition probability a_{jz}.* The estimated a_{jz} is denoted by $\overline{a}_{jz}$, for $j \neq z$, and is computed as the following:

$$\zeta(t,j,z) = f(t,j)e(t+1,z)b(t+1,z) \tag{27}$$

$$\log(\zeta(t,j,z)) = \log(f(t,j)) + \log(e(t+1,z)) + \log(b(t+1,z)) \tag{28}$$

$$\overline{a}_{jz} = \frac{\sum_{t=1}^{T-1}\zeta(t,j,z)}{\sum_{t=1}^{T-1}\gamma(t,j)} \tag{29}$$

2.4.7. *Estimate the emission parameters, an overview:* Because we fix c and R_m as constant, parameter estimation will only concern the negative binomially distributed component.

To estimate the negative binomial parameters, a weighted GLM function is implemented in C++. The argument of this function include “family”, “observation”, “covariate”, “offset”, and “prior”. The argument “family” means either Poisson or negative binomial. The argument “prior” means the probability that each observation belongs to the negative binomially distributed component. For the tth window and state j, the “prior” is denoted by $p_{t,j}$ and is computed as the following:

$$p_{t,j} = \frac{(1-c)e^{NB}(t,j)\gamma(t,j)}{c/R_m + (1-c)e^{NB}(t,j)} \tag{30}$$

Following the implementation function `MASS/glm.nb` in R [9], we use an alternating iterative estimation procedure to obtain the new estimate of $\log(\mu_{tj})$, denoted by $\log(\overline{\mu}_{tj})$, and the new estimate of ϕ, denoted by $\overline{\phi}$.

- First, we fix $\overline{\phi}$ and compute $\log(\overline{\mu}_{tj})$ by fitting weighted GLM using the iteratively reweighted least squares (IRLS) method. For details, see Section 2.4.8.
- Then, we fix $\log(\overline{\mu}_{tj})$ and compute $\overline{\phi}$ using the Newton-Raphson method with weight. For details, see Section 2.4.8.
- The above two steps alternated until convergence.

2.4.8. *Estimation of* $\log(\mu_{tj})$ *using the IRLS method.* ***Step 1.*** Define the necessary variables for estimating $\log(\mu_{tj})$, where $t = 1...T$, $CN_t = q_t = 0...j..(N-1)$.

- "Prior"
  $p_t = \{p_{t,0}, ..., p_{t,j}, ..., p_{t,N-1}\}$
  $p = \{p_1, ...p_t, .., p_T\}$
- "Observation"
  $y_t = \{o_t, ...o_t, ...o_t\}$ (o_t repeats for N times)
  $y = \{y_1, ...y_t, .., y_T\}$
- "Covariates"
  Let `cov` denote covariates and let M denote the number of covariates.
  If GC content (G) is the only covariate, $M = 1$ and define the covariate vector as:
  $x_t = \{g_t, ...g_t, .., g_t\}$ (g_t repeats for N times)
  $x = \{x_1, ...x_t, .., x_T\}$
  If $M > 1$, each covariate will be inserted into x like G
  $\texttt{cov}_t = \{\texttt{cov}_{t,0}, \texttt{cov}_{t,1}, ..., \texttt{cov}_{t,N-1}\}$
  $\texttt{cov} = \{\texttt{cov}_1, ...\texttt{cov}_t, .., \texttt{cov}_T\}$
  $x = \{\texttt{cov}^1, ..., \texttt{cov}^M\}$
- "Offset"
  $\text{offset}_t = \{[\log(CN_t = 0.5)+\log(l_t)], [\log(CN_t = 1)+\log(l_t)], ...[\log(CN_t = j)+\log(l_t)], .., [\log(CN_t = N-1) + \log(l_t)]\}$
  $\text{offset} = \{\text{offset}_1, ...\text{offset}_t, .., \text{offset}_T\}$
- "The weighted log-likelihood function"

$$Lm = \sum_{t=1}^{T}\sum_{j=0}^{N}\left[\log\left(\Gamma(o_t + 1/\phi)\right) - \left(\frac{1}{\phi} + o_t\right)\log(\frac{1}{\phi} + \mu_{tj}) + \log(o_t + 1.0) + o_t\log(\mu_{tj})\right] p_{tj} \tag{31}$$

Step 2. Fit a weighted Poisson regression model using the IRLS procedure.

Step 3. Perform a score test
The score test [4] is used to test whether the overdispersion parameter, ϕ, is significantly greater than 0. If the score test is significant, we

- Estimate ϕ using the Newton-Raphson method as described in Section 2.4.9.
- Proceed to Step 4.

Step 4. Fit a weighted negative binomial regression model using the IRLS method.

2.4.9. *Estimation of overdispersion using the Newton-Raphson method.* Given $\log(\mu_{tj})$, we use the Newton-Raphson method to estimate the overdispersion parameter, ϕ. In this study, we estimate one overdispersion parameter ϕ jointly for all states, and set $\phi_j = \phi$ for $j = 0..N-1$.

The following weighted log-likelihood and its first, second derivatives are used in the Newton-Raphson method to estimate ϕ. The weighted log-likelihood is the same as Equation 31.

$$Lm = \sum_{t=1}^{T}\sum_{j=0}^{N}\left[\log\left(\Gamma(o_t+1/\phi)\right) - \left(\frac{1}{\phi}+o_t\right)\log(\frac{1}{\phi}+\mu_{tj}) + \log(o_t+1.0) + o_t\log(\mu_{tj})\right]p_{tj}$$

It is computationally slightly easier to estimate $\varphi = 1/\phi$. Then,

$$Lm = \sum_{t=1}^{T}\sum_{j=0}^{N}\left[\log\left(\Gamma(o_t+\varphi)\right) - (\varphi+o_t)\log(\varphi+\mu_{tj}) + \log(o_t+1.0) + o_t\log(\mu_{tj})\right]p_{tj}$$

Thus the score function is

$$Score(\varphi) = \frac{\partial Lm}{\partial\varphi} = \sum_{t=1}^{T}\sum_{j=0}^{N-1}\left[\Psi(o_t+\varphi) - \Psi(\varphi) - \frac{\varphi+o_t}{\varphi+\mu_{tj}} - \log(\varphi+\mu_{tj}) + 1 + \log(\varphi)\right]p_{tj}$$

where $\Psi(x) = \partial\log\Gamma(x)/\partial x$, the digamma function. The observed Fisher information is

$$Info(\varphi) = -\frac{\partial^2 Lm}{\partial\varphi^2} = \sum_{t=1}^{T}\sum_{j=0}^{N-1}\left[-\psi(o_t+\varphi) + \psi(\varphi) + \frac{\mu_{tj}-o_t}{(\varphi+\mu_{tj})^2} + \frac{1}{\varphi+\mu_{tj}} - \frac{1}{\varphi}\right]p_{tj}$$

where $\psi(x) = \partial^2\log\Gamma(x)/\partial x^2$, the trigamma function.

We use the Newton-Raphson method given the score function and the fisher information. Initialize $\varphi = \frac{\sum_{t=1}^{T}\sum_{j=0}^{N-1} p_{tj}}{\sum_{t=1}^{T}\sum_{j=0}^{N-1} p_{tj}(o_t-\mu_{tj})^2}$

WHILE (ABS(Dev) > Toleration) {
  Dev = $\frac{Score(\varphi)}{Info(\varphi)}$
  $\varphi = \varphi$+Dev
}

2.5. **Post-segmentation Processing.** Following the discovery in [8], it is crucial to merge CNV calls and filter false positives. We applied the same procedure to merge CNV calls. In addition, we used a combination of read-depth accessible (RDA) filter and confidence score as used in [8] as the filter to remove low confidence calls.

References

[1] Goncalo R Abecasis, Adam Auton, Lisa D Brooks, Mark a DePristo, Richard M Durbin, Robert E Handsaker, Hyun Min Kang, Gabor T Marth, and Gil a McVean. An integrated map of genetic variation from 1,092 human genomes. *Nature*, 491(7422):56–65, November 2012.

[2] LE Baum, T Petrie, G Soules, and N Weiss. A maximization technique occurring in the statistical analysis of probabilistic functions of Markov chains. *The annals of mathematical statistics*, 1970.

[3] J A Bilmes. A gentle tutorial of the EM algorithm and its application to parameter estimation for Gaussian mixture and hidden Markov models, 1998.

[4] C B Dean. Testing for Overdispersion in Poisson and Binomial Regression Models. *Journal of the American Statistical Association*, 87(418):451–457, 1992.

[5] Jianqing Fan and Jinchi Lv. Nonconcave Penalized Likelihood With NP-Dimensionality. *IEEE Transactions on Information Theory*, 57(8):5467–5484, August 2011.

[6] Heng Li and Richard Durbin. Fast and accurate short read alignment with Burrows-Wheeler transform. *Bioinformatics (Oxford, England)*, 25(14):1754–60, July 2009.

[7] Ryan E Mills, Klaudia Walter, Chip Stewart, Robert E Handsaker, Ken Chen, Can Alkan, Alexej Abyzov, Seungtai Chris Yoon, Kai Ye, R Keira Cheetham, Asif Chinwalla, Donald F Conrad, Yutao Fu, Fabian Grubert, Iman Hajirasouliha, Fereydoun Hormozdiari, Lilia M Iakoucheva, Zamin Iqbal, Shuli Kang, Jeffrey M Kidd, Miriam K Konkel, Joshua Korn, Ekta Khurana, Deniz Kural, Hugo Y K Lam, Jing Leng, Ruiqiang Li, Yingrui Li, Chang-Yun Lin, Ruibang Luo, Xinmeng Jasmine Mu, James Nemesh, Heather E Peckham, Tobias Rausch, Aylwyn Scally, Xinghua Shi, Michael P Stromberg, Adrian M Stütz, Alexander Eckehart Urban, Jerilyn a Walker, Jiantao Wu, Yujun Zhang, Zhengdong D Zhang, Mark a Batzer, Li Ding, Gabor T Marth, Gil McVean, Jonathan Sebat, Michael Snyder, Jun Wang, Kenny Ye, Evan E Eichler, Mark B Gerstein, Matthew E Hurles, Charles Lee, Steven a McCarroll, and Jan O Korbel. Mapping copy number variation by population-scale genome sequencing. *Nature*, 470(7332):59–65, February 2011.

[8] Jin P Szatkiewicz, WeiBo Wang, Patrick F Sullivan, Wei Wang, and Wei Sun. Improving detection of copy-number variation by simultaneous bias correction and read-depth segmentation. *Nucleic acids research*, 41(3):1519–32, 2013.

[9] W N Venables and B D Ripley. Modern Applied Statistics with S Fourth edition by. *World*, 53(March):86, 2002.
